# Meiotic spindle assembly checkpoint and aneuploidy in males versus females

**DOI:** 10.1007/s00018-018-2986-6

**Published:** 2018-12-18

**Authors:** Simon Lane, Liisa Kauppi

**Affiliations:** 1 Department of Chemistry and the Institute for Life Sciences, University of Southampton, Building 85, Highfield Campus, Southampton, SO171BJ UK; 2 Faculty of Medicine, University of Helsinki, Haartmaninkatu 8, 00014 Helsinki, Finland

**Keywords:** Meiosis, Oocyte, Spermatocytes, Chromosome segregation, Aneuploidy, Recombination, Cohesin, Chiasma, Crossover

## Abstract

The production of gametes (sperm and eggs in mammals) involves two sequential cell divisions, meiosis I and meiosis II. In meiosis I, homologous chromosomes segregate to different daughter cells, and meiosis II resembles mitotic divisions in that sister chromatids separate. While in principle the process is identical in males and females, the time frame and susceptibility to chromosomal defects, including achiasmy and cohesion weakening, and the response to mis-segregating chromosomes are not. In this review, we compare and contrast meiotic spindle assembly checkpoint function and aneuploidy in the two sexes.

## Introduction

**Fig. 1:**
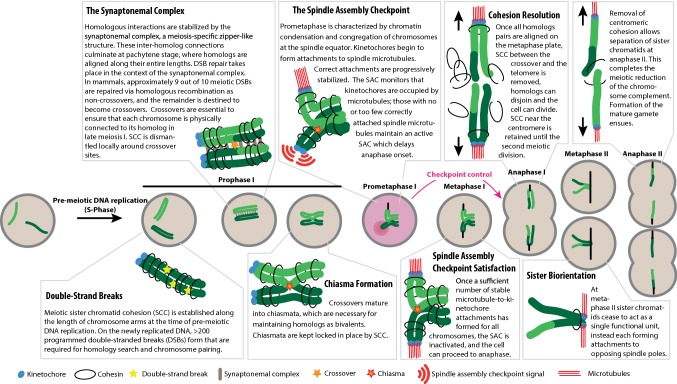
Key events of meiosis I

Life begins with the fusion of two haploid gametes. Gametes (eggs and sperm in mammals) are produced from diploid progenitor cells through a series of carefully orchestrated chromosomal events that include one round of DNA replication, followed by two rounds of cell division termed meiosis I and meiosis II (Fig. 1). During cell division, the spindle assembly checkpoint (SAC) is the guardian of faithful chromosome segregation. Compromised SAC function in meiosis can lead to the formation of aneuploid gametes that, in the vast majority of cases, are incompatible with subsequent development of the embryo, presumably because the incorrect complement of chromosomes leads to massive gene imbalances [1]. Permissible autosomal aneuploidies in humans invariably involve the gain of a chromosome (Box 1). Gamete aneuploidy often results in an inability of a couple to achieve pregnancy, or in increased chance of early pregnancy loss. Indeed, around one in seven couples now require assisted reproductive techniques to aid conception and pregnancy [2]. Most (84%) embryonic trisomies are found to derive from female meiosis, with errors in meiosis I contributing significantly more than meiosis II [3].

Aneuploidy arises from a two-step process that consists of the occurrence of a chromosome with the propensity to mis-segregate, followed by failure of the meiotic SAC to react to such a situation. In this review, we will look at both steps. Generally, oocytes fare poorly compared to spermatocytes when it comes to accuracy of chromosome segregation. Cells of the male and female germ line are on uneven playing fields, both with regard to chromosomal events leading up to the first meiotic division, as well as SAC function. Here, we compare the origins and fate of mis-segregating chromosomes in male and female mammals. Our emphasis will largely be on aneuploidy and the SAC in mice, where genetic studies have provided most of our current understanding of this topic. We also focus on the contributions of chromosomal events in prophase I to meiotic aneuploidy.

Box 1: Human aneuploidy syndromes There are several well-defined aneuploidy-associated clinical phenotypes in humans. It is noteworthy that aneuploid gametes are vastly (by several orders of magnitude) more common than live-born aneuploid individuals. Further, the incidence of aneuploidy syndromes does not reflect the propensity of particular chromosome pairs to mis-segregate but rather, the viability of abnormal karyotypes. When spontaneous abortuses are considered, aneuploidy of chromosome 16 is most common, but this chromosomal abnormality is not compatible with life. Even of the theoretically permissible aneuploidies (below), the vast majority do not come to term ([109, 110] and references therein).

Sex chromosome aneuploidies, Klinefelter syndrome (XXY, trisomy for sex chromosomes) and Turner syndrome (X0, monosomy for the X chromosome), are a common class of chromosomal abnormality in live-born individuals. Klinefelter syndrome affects 1 in 500–600 baby boys. Turner syndrome constitutes the only viable monosomy in humans. Autosomal monosomies in humans are embryonically lethal, presumably because the lowered gene dosage of any one complete autosome causes a huge imbalance in protein homeostasis. Autosomal trisomies that are sometimes viable are trisomy 21 (Down syndrome), trisomy 13 (Patau syndrome) and trisomy 18 (Edwards syndrome). Of these, Down syndrome is most common. Its incidence drastically increases with increased age of the mother.

## Key events in meiosis I

### Duration of meiosis I in males and females

**Fig. 2:**
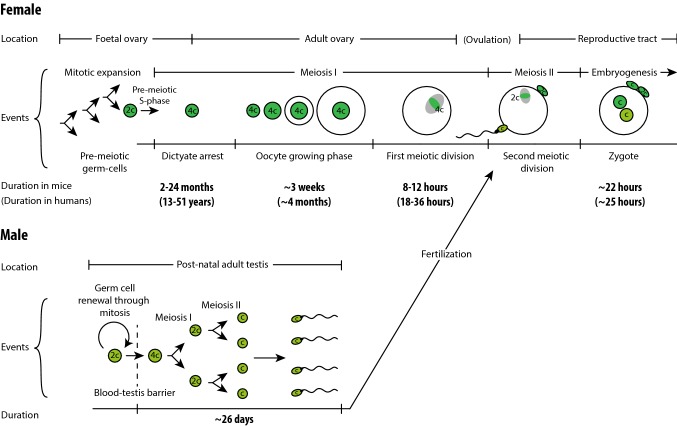
Female versus male meiosis. The duration of female meiosis is substantially longer than that of male meiosis, mainly due to the dictyate arrest that can last for years

Perhaps the most striking difference in the formation of female and male gametes is their developmental timescale (Fig. 2): meiosis I takes years in females, but less than 14 days in male mice [4]. Oocytes are created from mitotic divisions in the foetal ovary, entering meiosis I and arresting at diplotene of prophase. This arrest is referred to as the dictyate stage. Completion of meiosis (and maturation of the oocyte to make a fertilisable gamete) does not occur until the oocyte is recruited during an ovarian cycle. Recruitment takes place in any cycle between menarche and menopause; thus, in humans, oocytes can remain arrested in meiosis I between ~ 13 and ~ 51 years. Even in mice, the dictyate stage lasts for up to 2 years.

### Chromosome pairing

Despite the vastly different overall timescales, the key steps of male and female meiosis are essentially identical. A successful first meiotic division requires that homologous chromosomes find each other, pair, undergo recombination and then segregate to different daughter cells. An outline of this complex process is presented in Fig. 1. In pre-meiotic S phase, each chromosome is replicated and cohesion is established between sister chromatids [5, 6]. For an in-depth review of meiotic cohesin complexes, we refer readers to Brooker and Berkovitz [7]. Early in meiosis, several hundred programmed double-strand breaks (DSBs) are introduced into the genome that enable subsequent homology search [8, 9]. Only a subset of meiotic DSBs matures into crossovers, that is, into reciprocal genetic exchanges between non-sister chromatids.

At least one crossover per homolog pair (the “obligate crossover”) is necessary for guaranteeing accurate chromosome segregation subsequently [10]. For a detailed review of meiotic crossover control, see Gray and Cohen [11]. Most crossovers in mammals are cytologically marked by the protein MLH1, and its immunofluorescent detection is a standard tool for estimating crossover frequency and location. Subsequently, crossovers mature into chiasmata that link homologs until anaphase I. The configuration of interlinked homologs is referred to as the bivalent. Non-exchange chromosomes (those without a crossover) are called achiasmate chromosomes at this stage, and they can only exist as univalents.

### Chromosome segregation

One key difference for chromosome segregation between mitosis and meiosis I is that in the latter, homologous chromosomes—rather than sister chromatids—must segregate to opposite spindle poles. Each homolog consists of replicated sister chromatids, whose sister kinetochores in meiosis I should achieve mono-orientation (Fig. 1), that is, they should be attached to the same pole by their kinetochores.

In prometaphase, bivalents begin to congregate and align on the cell equator. At this time, they form attachments to spindle microtubules via their kinetochores. These microtubules are referred to as kinetochore fibres or k-fibres. Tension, which is critical for the progressive stabilization of k-fibres, is provided by chiasmata between the homologs. The SAC monitors microtubule-to-kinetochore attachments and controls the progression from metaphase to anaphase, as discussed in more detail below. Once the SAC is satisfied, the cell divides and homologs end up in different daughter cells.

## Crossover failure and/or cohesion loss generates abnormal chromosome configurations

Having described above the physiology of chromosome segregation, we now address how shortcomings in this process can lead to aneuploidy. As mentioned, aneuploidy requires that chromosomes be susceptible to mis-segregation, and that this be followed by a failure of the cell to identify or respond to those chromosomes. Here we discuss two ways in which abnormal chromosome configurations that predispose to aneuploidy may be created in the first meiotic division, namely non-exchange/achiasmy or premature cohesion loss.

### Non-exchange and/or achiasmate chromosomes

In both male and female mice, autosomes rarely suffer from lack of crossing-over [12–14]. However, recombination of male sex chromosomes and their subsequent segregation is challenging because the X and Y chromosomes are for a large part non-homologous [15]. This means that X–Y recombination, crossovers and chiasmata are spatially restricted to the small region of homology, the pseudoautosomal region (PAR) [16, 17]. Failure to form a DSB on the PAR will lead to non-exchange sex chromosomes [18] that, in turn, increase the chances of X–Y mis-segregation. Indeed, of all chromosomes, the X and Y are most frequently aneuploid in sperm. The vulnerability of the PAR to recombination failure appears to apply to humans as well (see also Boxes 1 and 3).

### Loss of arm and/or centromeric cohesion

Cohesion along the arms of the chromosomes is necessary for securing chiasmata in place. In females at least, crossovers that are located close to the telomere may “slide off” the ends of homologous chromosomes when they spend an extended period of time in this configuration and SCC weakens [19]. This can lead to premature separation of homologs, i.e. univalent formation [20].

In meiosis I, sister kinetochores are required to act as a single functional unit, ensuring separation of the homologs, but not the sister chromatids. Centromeric cohesion is essential for tethering sister chromatids together, and its loss results in greater separation of the sister kinetochores within each homolog of the bivalent [21–23]. Significant cohesion loss in the pericentromeric region permits separation of the two sister kinetochores to a point where they can start to behave independently [24]. This, in turn, enables aberrant bi-orientation of sisters.

**Fig. 3:**
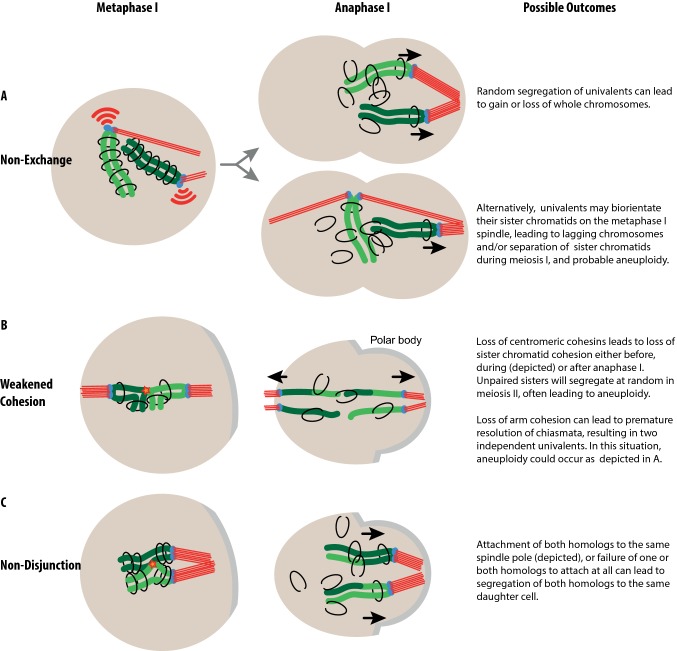
Meiosis I defects can result in aneuploidy. **a** Chromosomes without a crossover (non-exchange chromosomes) face problems at metaphase. They cannot be aligned with appropriate orientation on the cell equator because they lack the physical tether required for generating inter-homolog tension. This leads to random segregation at anaphase and a high likelihood of aneuploidy. **b** Weakened cohesion can result in the destabilization of the bivalent configuration of homologous chromosomes. Some reports suggest univalents are formed in metaphase I, leading to segregation akin to **a**. In the example shown here, premature loss of centromeric cohesion allows for the premature resolution of sister chromatids. **c** Non-disjunction, as is often observed in young mouse oocytes. Here, a failure of the homologous chromosomes to establish bi-orientation leads to their co-segregation to one spindle pole, resulting in loss or gain of chromosomes

To summarize, failure to form a crossover on the PAR (for male mice) and cohesion loss (for female mice) are major contributors in generating chromosomes that are prone to mis-segregation at anaphase (Fig. 3). These paths to aneuploidy are also interlinked—when cohesion weakens, chiasmata are lost and sister kinetochores begin to separate [22, 23]. Premature cohesion loss and achiasmy both generate defective chromosome configurations that the meiotic SAC should monitor during prometaphase and metaphase. Next, we will discuss this central cellular surveillance mechanism that keeps such errors in check.

## The spindle assembly checkpoint

Aneuploidy in the germ line should only occur if the surveillance mechanisms employed to detect chromosomes at risk for mis-segregation are ineffective. In this section, we discuss the spindle assembly checkpoint (SAC). By way of introduction, we first tackle the SAC in mitosis, where it has been best studied.

Central to SAC function are the kinetochore, where the SAC is located and where chromosome-spindle attachments are monitored, and the anaphase-promoting complex (APC), the downstream target of the SAC that drives cells irreversibly from metaphase to anaphase. The process of assembling the spindle and then correctly aligning all chromosomes in a bi-oriented fashion at the spindle equator is somewhat stochastic, and the time required to achieve this will vary from one division to the next [25]. The SAC, therefore, must be able to control cell cycle progression, only permitting advancement to anaphase when all chromosomes have formed correct attachments to the spindle. The SAC, therefore, inhibits the APC throughout prometaphase, only releasing the inhibition at metaphase, when satisfied by correct kinetochore–microtubule attachments. The APC then initiates the metaphase-to-anaphase transition by orchestrating proteolytic destruction of key proteins Cyclin B1 and securin [26].

The core of the SAC is the mitotic checkpoint complex (MCC) which acts to inhibit the APC. The MCC consists of the proteins MAD2, BUBR1, BUB3 and the APC activator CDC20. Other SAC proteins include MAD1 which is key to catalysing MCC formation at kinetochores, BUB1 which is required for the hierarchical recruitment of other SAC proteins to kinetochores, and MPS1 which is considered the master kinase of the checkpoint. MPS1 phosphorylation of the outer kinetochore protein KNL1 occurs in a microtubule-binding-dependent manner, and thus allows the SAC to be responsive to attachment status of individual kinetochores. MPS1 phosphorylation sites on the outer kinetochore proteins form the most upstream sites of recruitment of the aforementioned SAC proteins. Lastly, the kinase Aurora B has a role in recruiting MPS1 to the kinetochore, in addition to roles in tension sensing and in promoting correction of erroneous microtubule–kinetochore attachments [27].

Activation of the SAC at a kinetochore causes hierarchical recruitment of proteins to the outer kinetochore. Central to MCC formation is a MAD1 homodimer bound to MAD2, which acts as the catalytic unit, incorporating further molecules of MAD2 and CDC20 with preformed BUB3 and BUBR1 to make the MCC. The MCC is then free to diffuse from the kinetochore and inhibit the APC throughout the cell (in the case of oocytes, across an unusually large cellular volume, see Box 2). An important facet of the SAC is that to achieve fidelity in chromosome segregation, the signal from only a single kinetochore must be sufficiently strong to inhibit the APC throughout the entire cell. For a detailed review of the molecular workings of the SAC, we refer the reader to [28]. The SAC is highly conserved across eukaryotes and appears to be very efficient (errors are estimated to occur only 1 in ~ 4000 mitoses [29]).

Box 2: Oocyte volume The SAC’s ability to respond to errors is thought to depend on the ratio of signalling kinetochores to cytoplasmic volume. Mouse oocytes are some 200 times larger than typical somatic cells [111], making this ratio pertinent. Several papers have now addressed this issue in mouse oocytes [49, 112, 113] as well as in Xenopus egg extract [114] and Caenorhabditis elegans early embryos [115].

Xenopus eggs are around 1mm in diameter and lack a spindle assembly checkpoint [116]. Interestingly, they do not display aneuploidy, suggesting another mechanism is at play to ensure correct chromosome segregation [117]. Xenopus egg extracts have the ability to periodically enter and depart from a mitotic state, even in the absence of DNA replication and division. Addition of sperm nuclei and the microtubule de-polymerising drug nocodazole could halt this cyclic progression, at concentrations of 9000 sperm nuclei per µL, but not at 4500 nuclei/µL [114]. Intriguingly, the volume per nucleus at which the checkpoint becomes apparent (active, 111 pL; not-active, ~ 222 pL) is in the same ballpark as a mouse oocyte (~ 200 pL), but orders of magnitude lower than in the Xenopus egg (0.52 µL). The number of signalling kinetochores per nuclei is also similar.

Galli and Morgan found a strong relationship between mitotic arrest time and kinetochore-to-volume ratio in C. elegans [115]. This relationship held true when either cell volume or kinetochore numbers were manipulated. In mice, the relationship between oocyte cytoplasmic volume and checkpoint strength has now been investigated in detail. Kyogoku and Kitajima used mouse oocytes, either doubled or halved in volume, and showed a relationship between volume and checkpoint stringency [113]. Smaller oocytes gained the ability to delay anaphase in response to non-alignment, with two non-aligned bivalents being the apparent threshold. This increased ability was only apparent, however, if oocyte volume was halved prior to nuclear envelope breakdown (NEB) indicating that the added stringency was not due to volume alone. Their work showed it was likely due to increased concentrations of the SAC proteins, which accumulate on the nuclear pores in prophase and then become diluted by the cytoplasm at NEB. Lane and Jones only investigated the effect of reduced volume and only after NEB [118]. Consistent with the work of Kyogoku and Kitajima, they found no ability of the oocyte to implement the checkpoint, even when volume was reduced to one eighth. Further, live imaging following spindle disruption with nocodazole in these small oocytes showed that even many non-aligned chromosomes could not prevent anaphase. Hoffmann and colleagues investigated halving oocyte volume after NEB and found that a single chromosome could delay APC activity in prometaphase [112], but whether it could prevent anaphase in response to non-alignment is not clear. In summary, it seems that the large volume of oocytes is not solely responsible for reduced SAC stringency, and that concentrations of SAC proteins available to kinetochores in meiosis I may be a more important factor.

## The SAC in meiosis I

Most SAC proteins studied behave similarly in mitosis and in meiosis of both sexes [30]. MAD2’s meiotic localization has been studied most extensively [31]. In mitosis, its association with the kinetochore is transient and is lost once stable microtubule attachments have been formed; this is also the localization pattern in meiosis I oocytes [31]. Interestingly, immunofluorescent localization experiments on rat and mouse spermatocytes (but not oocytes, see also [32]) showed that MAD2 remains on kinetochores throughout the entire first meiotic division [31]. A possible explanation is that kinetochores of first-division spermatocytes never experience full microtubule occupancy [31] but the significance of this remains unclear. As expected, unaligned meiosis I homolog pairs showed substantially brighter MAD2 kinetochore signals.

**Fig. 4:**
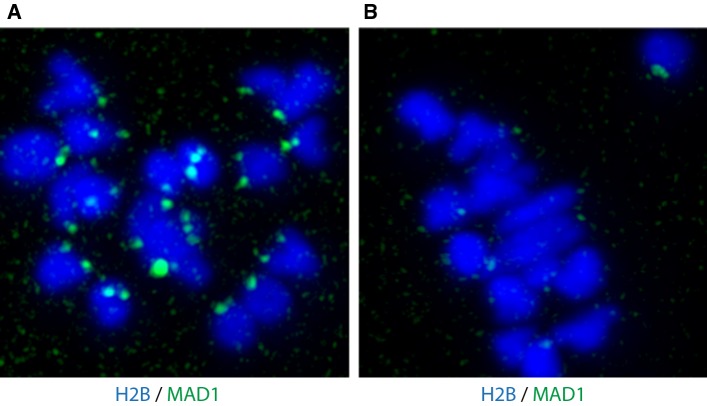
SAC proteins localize to meiotic kinetochores. MAD1 localizes to kinetochores of a mouse oocyte expressing MAD1-2GFP (green) and H2B-mCherry (blue) in prometaphase (**a**) and metaphase (**b**) of meiosis I. Note the non-aligned chromosome in **b** recruiting modest levels of MAD1. Modified from Ref. [35]

In addition to MAD2, MAD1 decorates prometaphase kinetochores in mouse oocytes and both proteins are recruited to unaligned chromosomes at metaphase ([33–35] and Fig. 4). Moreover, MPS1 [36], BUBR1 [37], BUB1 [38] and BUB3 [39] localize to oocyte kinetochores/centromeres in mice; BUB1 and BUBR1 have been shown to correctly localize in human oocytes [40]. SAC proteins have not been found to behave differently in female meiosis I when compared to mitosis, excepting that events take place over much longer timescales in oocytes. One notable deviation from mitosis, however, is the meiotic expression of Aurora kinase variant Aurora C, which has high sequence similarity with Aurora B. The two appear to have somewhat overlapping roles during meiosis I, with Aurora C localizing to chromosomes like Aurora B but with additional functionality, e.g. localizing to spindle poles like Aurora A [41, 42]. Both Aurora B and C bind to the chromosomal passenger complex, and Aurora C can completely compensate for Aurora B loss in mitosis [43]. Aurora C is more stable in oocytes than aurora B and may be the dominant Aurora kinase [44, 45].

Aurora kinases are central to the debate (in mitosis and meiosis) as to whether the SAC detects tension across kinetochores (or chromosomes), or whether it is sensitive to microtubule attachment status only. Since loss of tension results in Aurora kinase-dependent loss of attachment, the two possibilities are not trivial to unravel. This has recently been addressed in oocytes, where it was found that Aurora kinase microtubule attachment is sufficient for SAC satisfaction, whilst lack of tension across bivalents is important for subsequent error correction [46].

## Lower levels of some, but not all, SAC proteins result in meiotic aneuploidy

In oocytes a “baseline” rate of non-disjunction, in the absence of any structural changes to the homologs, exists at around 3–4% even in young mice, and thus appears to be age independent [47–50]. In older mice, the error rate increases, presumably as abnormal chromosome configurations begin to factor in. This age-decline trend, seen in both mice and humans [48, 51], is consistent with a loss of cohesion during the protracted prophase arrest (see “Age-related cohesion loss”).

In mitotically dividing cells in vivo, heterozygosity of many SAC genes can lead to accelerated progression into anaphase and to aneuploidy (see, e.g. [52] and references therein). Wild-type SAC protein levels, therefore, seem to be critical for normal SAC stringency.

Meiotic studies in males are limited to reducing SAC protein dosage genetically and then examining chromosome segregation outcomes in spermatocytes or sperm. Interestingly, genes whose heterozygosity produced aneuploid splenocytes (*Bub3*, *Rae1*, *Bub3*/*Rae1* double heterozygotes and *Rae1*/*Nup98* double heterozygotes) did not show such effect in spermatocytes [52]. Thus far, the only mouse models where reduced SAC protein levels have been shown to result in lower SAC fidelity in male meiosis are *BubR1* hypomorphs [53] and *Mad2* heterozygotes [54] (Table 1).

**Table 1:** Consequences of compromised SAC protein function in meiosis

Gene	Setting	Gender	Consequence	References
*Mad2*	In vivo (*Mad2*^+/−)^	F	Accelerated meiotic progression and meiosis I chromosome mis-segregation in > 20% of oocytes	[124]
	In vivo (*Mad2*^+/−^)	M	Low-level sperm aneuploidy in the presence of non-exchange sex chromosomes	[49]
	Microinjection of dominant-negative Mad2 into oocytes	F	Premature anaphase onset	[32]
	Depletion in oocytes using morpholino	F	Increased aneuploidy, premature degradation of cyclin B and securin, accelerated meiosis I progression	[50]
*Bubr1*	In vivo (*Bubr1*^+/−^)	F	Unstable microtubule-to-kinetochore attachments, relaxed SAC	[37, 125]
	In vivo (*Bubr1*^+/−^)	M	No effect reported	[125]
	In vivo (hypomorph *Bubr1*^H/H^)	M	Elevated aneuploidy in secondary spermatocytes	[125]
*Bub1*	Oocyte-specific conditional KO	F	Accelerated chiasma resolution, PSSC, bivalent malorientation, chromosome mis-segregation	[126]
	In vivo (*Bub1*^+/−^)	F	Meiosis I aneuploidy and PSSC in oocytes; males: no effect	[127]
	In vivo (*Bub1*^+/−^)	M	No effect reported	[127]
*Bub3*	RNAi in oocytes	F	Chromosome misalignment, aneuploidy	[39]
*Mps1*	Oocyte-specific conditional KO	F	MAD2 fails to localize to kinetochores, premature APC activation, 70% of meiosis II oocytes aneuploid	[36]
*AurkB/C*	Oocyte-specific conditional ATP-binding pocket mutant of AURKC	F	Most oocytes arrest at metaphase I, escapees are aneuploid	[41]
	In vivo (*Aurkc*^−/−^)	F	Increased metaphase I arrest, increased chromosome misalignment, aneuploidy not increased	[44]
	In vivo (*Aurkc*^−/−^)	M	No effect on meiosis I, subfertility due to post-meiotic spermatogenic defects	[128]
	Spermatocyte-specific conditional KO of *Aurkb*	M	Metaphase I arrest and apoptosis	[128]
	Microinjection of kinase-dead Aurkc into oocytes	F	Misaligned chromosomes, premature chromosome segregation, abnormal k-fibre attachments, BUB1 and BUBR1 fail to localize to kinetochores	[129]
	Aurora kinase inhibitor	F	Frequent chromosome misalignment, accelerated meiotic progression, premature anaphase onset	[130]

*F* female, *M* male

Thanks to oocyte culture, experimental approaches to manipulate SAC function in female meiosis are more varied. In addition to genetic studies, RNAi and morpholinos can be utilized. In contrast to spermatocytes, effectively all investigations concluded that reducing the function of a SAC component results in measurable chromosomal defects and/or aneuploidy in oocytes (Table 1). SAC function in males, therefore, appears more robust compared to females.

## SAC sensitivity in oocytes versus spermatocytes

In addition to manipulating SAC components themselves, differences in SAC function in male versus female meiosis I can be revealed using mouse models with increased frequencies of abnormal chromosomes that ought to activate the SAC. One example is the *Mlh1*^−/−^ mouse where almost no meiotic crossovers form, resulting in univalents in meiosis I (Fig. 3a). In spermatocytes, this leads to metaphase arrest, apoptosis and subsequently, infertility [55]. Although *Mlh1*^−/−^ females are also infertile, their oocytes can occasionally progress through meiosis I to extrude a polar body [55, 56], indicating that the SAC in *Mlh1*^−*/*−^ females is more permissive than in males. Male mice carrying Robertsonian fusion chromosomes (fusions of two telocentric chromosomes to form a single metacentric one) have increased numbers of unpaired and non-aligned chromosomes in meiosis I and show increased metaphase I arrest and apoptosis in spermatocytes [57]. This is again consistent with a SAC response, albeit an incomplete one, as aneuploid sperm are also formed [57]. In females, single Robertsonian fusions do not appear to cause metaphase arrest [58]. In *Sycp3* knockout females, modest numbers of univalents are produced, and these fail to trigger a checkpoint arrest in meiosis I [59]. The XO mouse, where the X chromosome lacks a homolog, is an example where only a single aberrantly behaving chromosome is present in oocytes [60, 61]. Oocytes do not arrest in metaphase I but have high rates of polar body extrusion. In contrast, in mice genetically engineered to lack a Y chromosome, the unpaired X chromosome triggers a robust metaphase I arrest in spermatocytes [62]. One caveat with this comparison is that the type of attachment between the univalent and the spindle, which has implications for detection by the SAC, is unclear. This is discussed further below (see “Univalents with bi-polar attachments can evade the SAC”).

The conclusion is that the female SAC can detect aberrantly behaving chromosomes only when they are present in high numbers, whereas in males, SAC sensitivity may be set at the level of a single chromosome, as is the case in mitosis. Next, we will examine factors that contribute to SAC leakiness in oocytes.

## The leaky SAC in oocytes

In oocytes, SAC proteins localize as expected (see above), and interfering with their function leads to aneuploidy. On the other hand, multiple lines of investigation have shown that SAC stringency in oocytes is low. How can these observations be reconciled? In the last decade, improvements in live cell imaging of oocytes have made it possible to discover the origins of mis-segregation by direct observation in vitro.

Detailed 3D time-lapse tracking of kinetochores in mouse oocytes demonstrated that initial attachments of bivalents to the meiotic spindle are inherently unstable [63]. Chromosomes often failed to make stable attachments at all; unstable attachments were unable to activate the SAC in both wild-type oocytes [64, 65] and in oocytes with disrupted spindle architecture [66, 67]. In these studies, SAC proteins were found to be recruited to the kinetochores of non-aligned chromosomes, consistent with a functional checkpoint, yet at the same time APC activity was not inhibited. Thus, the SAC in oocytes responds to errors but is unable to provide the power required to prevent anaphase. When SAC strength was measured (by its ability to prevent APC activity) during meiosis I, it was found to be partially active, even during metaphase when chromosomes were aligned. This activity contributes a significant delay to the timing of anaphase and reduces aneuploidy rates [68]. The SAC, therefore, seems to function more as a timer or gentle brake in female meiosis I, rather than a strict checkpoint. This limited checkpoint activity is consistent with observations in mitotic cells that checkpoint response can be graded [69]. An interesting facet of the SAC in female meiosis I is its ability to generate a strong checkpoint in response to DNA damage [35, 70–72], demonstrating that the oocyte is capable of complete APC inhibition. Why this power cannot be brought to bear in response to chromosome attachment errors requires further investigation.

### Large oocyte volume can dampen SAC signalling

Compared with spermatocytes, SAC function in oocytes appears more dependent on wild-type protein levels (Table 1). Because protein levels vary cell-to-cell, in any given oocyte there may be a SAC-associated component whose local concentration is below a critical threshold for ensuring normal SAC function. This effect is likely exacerbated by the unusually large volume of oocytes (Box 2). Similarly, the kinetochore-to-volume ratio in oocytes is some orders of magnitude less than in a typical somatic cell. This may explain the SAC’s inability to fully restrain the APC when the wait-anaphase signal emanates from a small number of kinetochores. Recent work has addressed the issue of cell volume in the oocyte and early embryo (Box 2).

### Univalents with bi-polar attachments can evade the SAC

Univalents may bi-orient their sister kinetochores and, subsequently, segregate sister chromatids at meiosis I. This type of “predivision”, i.e. premature division of meiosis I kinetochores, was first described for human oocytes [73]. Predivision can be either balanced, such that both univalent homologs separate their sisters into different daughter cells, or unbalanced, in which case one homolog undergoes sister chromatid separation while the other does not (Fig. 3a, bottom). Alternatively, sister kinetochores can be maintained as a functional unit, with both attaching to a single spindle pole as is normal for meiosis I. This arrangement may be more likely to generate a non-disjunction outcome (Fig. 3a, top).

The type of attachments formed by the univalents may have implications for their detection by the SAC, although in females it is likely that either arrangement falls below the threshold for detection. Univalent bi-orientation would be expected to satisfy the SAC, as tension is generated across the univalent, leading to stable attachment [59]. However, when sister kinetochores are maintained as a functional unit, they would not be expected to generate stable attachment, and should be more likely to promote SAC signalling. Studies in the XO mouse suggest that at anaphase, ~ 40% of univalents undergo predivision whilst ~ 60% are segregated to one spindle pole, inferring a reasonably even split between the two modes of attachment [74]. Mechanistic insight into this process was recently provided by high-resolution imaging of aged live mouse oocytes [75]. In a subset of aged oocytes, bivalents first separated into univalents, followed by unscheduled sister chromatid separation in meiosis I, favouring a view where univalents bi-orientate, and are thereby likely to evade the SAC.

Abnormal bi-polar attachment of univalents is likely relevant for human females as well. Using MeioMapping to track genetic markers in human oocytes and polar bodies, Ottolini et al. recently identified a novel pattern of chromosome segregation that they termed reverse segregation [76]. This refers to a situation where both homologs undergo sister chromatid separation at meiosis I. Chromosome arrangements that could facilitate such a division have been observed at metaphase in human oocytes [77]. Reverse segregation lends support to the idea that the SAC in human females, as in mice, tolerates unusual meiosis I chromosome configurations, at least in the IVF-treated population.

The above-cited studies and others have demonstrated that in female meiosis I, univalents can escape SAC surveillance, essentially by behaving like mitotic chromosomes. Whether or not univalents in males could potentially utilize the same strategy is not clear. The general consensus that the SAC in female meiosis is leaky, but against this backdrop, it should be appreciated that the meiotic SAC in males is not especially stringent either, as we shall discuss next (see also [78]).

## Evidence for SAC leakiness in males

Compared to females, and aged females in particular (see below), male mice are able to segregate meiotic chromosomes quite accurately. This, we propose, is due to a low incidence of chromosomes prone to mis-segregation in the first place, rather than high SAC fidelity.

A stringent SAC response should prevent the formation of aneuploid daughter cells. This is not exactly true in male meiosis, as demonstrated by the following examples. First, univalents in Robertsonian translocation mice frequently evade that SAC, resulting in aneuploid sperm [57], as already mentioned. Second, in vivo treatment with nocodazole, a microtubule-disrupting chemotherapeutic agent, delays meiotic progression but ultimately a fraction of spermatocytes divides with non-disjoined chromosomes, forming disomic sperm [79]. Moreover, inter-strain differences in the frequency of aneuploid sperm testify to relaxed SAC stringency. For example, sperm aneuploidy in PL/J mice is substantially higher than in C57BL/6 mice [80]. In XYSxr mice on a mixed C3H/HeH-101/H strain background, more than 10% of spermatocytes with non-exchange X–Y chromosomes can escape the SAC (calculated from data presented in [81]). The fact that under all these conditions, aneuploid sperm are produced—albeit at variable frequencies—implies that the male SAC is leakier than perhaps generally appreciated. This effect may simply be masked by the low incidence of misbehaving homolog pairs in most wild-type spermatocytes.

## Genetic background matters for fidelity of chromosome segregation

In addition to the variability in SAC stringency and sperm aneuploidy between different strains of laboratory mice (above), there is evidence that genetic background substantially affects chromosome segregation outcomes in females as well. The ability of the univalent X chromosome in XO mice to bi-orientate depends to a degree on strain background [74]. Strain background also has a major impact on age-related oocyte aneuploidy: the most commonly used laboratory strain C57BL/6 appears relatively resistant to age-related aneuploidy and to loss of the cohesin protector SGO2 when compared to the Swiss CD-1 strain [82].

No doubt there are numerous modifying factors responsible for this variation, even in the presence of a “wild-type”, i.e. unperturbed SAC. How much of this variation can be explained by inter-strain differences in critical SAC protein levels remains to be investigated. For instance, strain-specific polymorphisms in regulatory regions of key SAC genes could lead to substantial changes in SAC protein levels, thus leading to altered SAC stringency. Similar processes are likely responsible for inter-individual variation in sperm aneuploidy in humans (Box 3) as well.

Box 3: Sperm aneuploidy in men Meiotic chromosome configurations and sperm aneuploidy in normal and infertile men have been assessed in many studies. The percentage of metaphase cells with aberrant chromosome configurations is surprisingly high, 15–16%, and practically all abnormalities involve the X and Y chromosomes [119]. Therefore, as in mice, the X and Y in humans are the weak point for meiotic chromosome segregation.

Aneuploid sperm are relatively common (4%) in normal control males [120]. Individuals with defects in X–Y pairing often also produce aneuploid sperm [121–123]. Meiotic SAC surveillance in men thus appears inefficient compared to mice. In men undergoing infertility treatment, substantial inter-individual variability was reported both in the inherent propensity to X–Y recombination defects, as well as the stringency with which meiocytes defective for sex chromosome recombination are eliminated [123].

## Age-related cohesion loss and aneuploidy

In females, the extended period of prophase arrest in oocytes places extraordinary demands on sister chromatid cohesion. The cohesive ties that hold chromatids and chromosomes together are susceptible to gradual loss over the female reproductive lifespan, in both mice and humans [22, 23, 83, 84]. In males, maintenance of sister chromatid cohesion is not a weak point for chromosome segregation due to the short duration of meiosis I (Fig. 2).

The half-life of typical proteins is estimated to be only a few days in mice and humans [85], although some, notably in the brain, have been found to be stable for up to a year [86]. Cohesin cannot be loaded onto chromosome arms during dictyate arrest or during oocyte growth in mice [87, 88], and loss of cohesion protein expression after oocytes enter meiotic arrest is not detrimental to fertility [89], together indicating non-renewal of cohesin after its initial loading in pre-meiotic S phase. In contrast, in *Drosophila* cohesion replenishment during meiosis I is required to prevent aneuploidy [90]. Further, aneuploidy was increased in mice with haploinsufficient cohesion proteins [91], indicating sensitivity of the oocyte to levels of these proteins before entering arrest. Maintenance of cohesion, therefore, probably relies on the exceptional longevity of cohesin ring proteins loaded during S-phase, far longer than is typical of other proteins, and not on renewal during the long period of arrest. Interestingly, age-related loss of cohesion may also be promoted indirectly by increased separase activity during interkinesis between meiosis I and II, in turn stemming from greater securin loss during meiosis I in oocytes from older mice [92]. This is consistent with other observations in aged oocytes, where formation of single sister chromatids is observed after completion of MI during interkinesis [82] and the incidence of single chromatids in MII is more frequent than that of univalents in MI [22].

Unscheduled cohesin loss can lead to aneuploidy through the most direct route, premature release of the homologous chromosomes from one another in meiosis I, that is, the bivalent falling apart into univalents, or by the premature release of sisters in meiosis I ([75], Fig. 3) or II. However, aneuploidy can also be generated by more subtle changes in chromosome structure arising from only partial loss of cohesion. This can be measured as increased distance between sister kinetochores within each kinetochore of a bivalent, thought to be indicative of loss of cohesion leading to increased flexibility within the centromeric region [23]. In turn, this may cause the establishment of erroneous k-fibre attachments to the meiotic spindle, leading to increased chance of chromosome mis-segregation at anaphase and subsequently to aneuploidy [24, 75, 77].

When aberrant chromosome configurations are present, it then falls to the SAC to detect them and to prevent progression to MII. However, as discussed above in the case of univalents in MI, if erroneous attachments sufficiently occupy the kinetochores and generate enough tension to be stabilized, they will likely satisfy the SAC. Still, there is evidence that the SAC is weaker in aged oocytes: when aged oocytes were challenged with nocodazole they were more likely to complete meiosis I than young oocytes. This may be explained by lower kinetochore levels of MAD2 and phosphorylated Aurora C, preventing the checkpoint from responding to attachment errors [82]. Lower levels of BUB1 and BUBR1 were also found on the kinetochores of aged human oocytes [40]. Increased APC activity in aged oocytes adds further weight to the notion that the SAC’s ability to limit progression to meiosis II decreases with age [92].

There is some indication of age-dependent increase in sperm aneuploidy in mice, but this mostly stems from meiosis II, not meiosis I [93]. Also in human males, an association between increased age and sperm aneuploidy was noted [94, 95]. Jeganathan and van Deursen observed an increase in aneuploid secondary spermatocytes of wild-type mice older than 2 years (3–7% of cells, compared to 0% in 5-month-olds [52]), indicating propensity to errors in meiosis I of older males as well. Koehler et al. reported no change in crossing-over in aging male mice [13]. More recently, crossover levels were actually found to increase with age; univalency of sex chromosomes and small autosomes also increased [96], implying overall mis-regulated crossover control. Intriguingly, in juvenile males, crossover assurance is less stringent, with reduced crossover levels and more non-exchange homolog pairs compared to adults [96, 97]. At present, there are no studies that pinpoint the mechanism(s) responsible for increased sperm aneuploidy with age. It seems plausible that a key factor is a decline in SAC stringency.

In contrast to the at best modest effects in older men, in women above the age of 35, gamete aneuploidy increases sharply. Maternal age remains the strongest associated risk factor overall for aneuploidy in embryos. Indeed, almost half of oocytes from IVF-treated women over the age of 38 are aneuploid [98]. Drivers of this aneuploidy include cohesin loss, which is likely the major age-related factor, and (age-independent) SAC function deficits, which are compounded by difficulties in bivalent bi-orientation on the meiotic spindle. It is worth noting that aneuploidy rates are surprisingly high even in oocytes of young women. Inefficient crossover maturation and subsequent achiasmy in human females [99] are age-independent factors that help explain this. On the other hand, oocytes with increased recombination are enriched in successful pregnancies in older women [76, 100], thereby counteracting crossover control defects [99, 100] in the overall oocyte pool.

## Environmental factors

The above settings, most of them created in laboratory mice in vivo or ex vivo, have provided profound insights into meiotic chromosome segregation and/or SAC fidelity. Some, such as reduced SAC protein levels (possibly via, e.g. miRNA-mediated downregulation, as demonstrated for mitotic cells [101]), likely apply to humans also. However, perhaps biologically the most relevant scenario to consider is how environmental factors may predispose to meiotic aneuploidy.

Many studies have assessed the effects of mutagen exposure on meiotic chromosome segregation. In males, there is no clear consensus about the identity of true aneugens. Exposure to chemotherapeutic agents has a transient effect on sperm aneuploidy [79, 102], with important implications for reproductive counselling of men undergoing cancer treatment. Alcohol and caffeine intake have also been reported to increase sperm aneuploidy [95]. A modest increase in sperm aneuploidy was reported in pesticide factory workers [103]. Exposing mice to cigarette smoke causes spindle abnormalities in oocytes [104], but whether this actually leads to aneuploidy is not clear.

To date, the most straightforward aneugenic effect is induction of oocyte aneuploidy by bisphenol A (BPA), a component of plastics and epoxy resins [105]. More recently, it was also shown that BPA leads to reduced crossing-over in spermatocytes. This effect was indirect, however, via exposure of spermatogonial stem cells to BPA [106] and it is not known whether these reduced crossover levels subsequently result in sperm aneuploidy. Non-ionizing radiofrequency radiation, emitted by mobile phones, seems to increase reactive oxygen species and DNA damage in sperm (reviewed in [107]) but no data exist on its possible effect on the fidelity of chromosome segregation. Since this environmental factor is now ubiquitous in developed countries, studies assessing germ cell aneuploidy after long-term exposure would be of major interest in the future.

## Concluding remarks

Over more than two decades, mouse genetics has provided fundamental insights into the molecular mechanisms of SAC function and chromosome segregation in male and female meiosis. Recent advances in high-resolution microscopy (mice) and high-throughput sequencing technology (humans) have unravelled how univalent chromosomes in females can escape meiotic SAC surveillance and how cohesion declines irreversibly with age. Studies of male meiosis have been limited by the inability to manipulate these biological processes ex vivo. With the advent of testicular fragment culture (now successfully used in meiotic recombination studies by Pacheco et al. [108]), it will be possible to expose spermatocytes to, e.g. small molecule inhibitors of SAC components, akin to experiments in oocytes. Combining these approaches will continue to elucidate the fascinating similarities and differences in meiotic chromosome segregation between males and females.

## Abbreviations

APC: Anaphase-promoting complex
BPA: Bisphenol A
DSB: Double-strand break
NEB: Nuclear envelope breakdown
MCC: Mitotic checkpoint complex
PAR: Pseudoautosomal region
PSSC: Premature separation of sister chromatids
RNAi: RNA interference
SAC: Spindle assembly checkpoint
SCC: Sister chromatid cohesion
